# Characterization of a pH-Tolerant Strain *Cobetia* sp. SASS1 and Its Phenol Degradation Performance Under Salinity Condition

**DOI:** 10.3389/fmicb.2019.02034

**Published:** 2019-09-04

**Authors:** Rongwu Mei, Meng Zhou, Luning Xu, Yu Zhang, Xiaomei Su

**Affiliations:** ^1^Environmental Science Research and Design Institute of Zhejiang Province, Hangzhou, China; ^2^College of Geography and Environmental Science, Zhejiang Normal University, Jinhua, China

**Keywords:** phenol degradation, acid mine drainage, high salinity, *Cobetia* sp., mineralization pathway

## Abstract

Biological treatment of complex saline phenolic wastewater remains a great challenge due to the low activity of bacterial populations under stressful conditions. Acid mine drainage (AMD) as a typically extreme environment, shaped unique AMD microbial communities. Microorganisms survived in the AMD environment have evolved various mechanisms of resistance to low pH, high salinity and toxic heavy metals. The primary goal of this work was to determine whether a strain isolated from an AMD could degrade phenol under stressful conditions such as low pH, high salinity and heavy metals. The results suggested that the strain *Cobetia* sp. SASS1 isolated from AMD presented different physiological characteristics in comparison with five most closely related species. SASS1 can efficiently degrade phenol at wide ranges of pH (3.0–9.0) and NaCl concentration (0–40 g/L), as well as the existence of Cu^2+^ and Mn^2+^. Specifically, the SASS1 could completely degrade 1500 mg/L phenol in 80 h at 10 g/L NaCl. Meanwhile, mineralization of phenol was achieved with complete degradation of 900 mg/L phenol and simultaneously COD decreasing from 2239 mg/L to 181.6 mg/L in 36 h. Based on biodegradation metabolites identification and enzyme activities analysis, both *ortho*-cleavage pathway and benzoic acid pathway for phenol degradation were proposed. These findings suggested that SASS1 was an efficient phenol degrader under salinity and acidic conditions, and could be considered as key population for bioremediation of industrial phenolic wastewaters under stressful conditions.

## Introduction

Phenolic compounds which are ubiquitous in industrial wastewater, are hazardous and carcinogenic owing to its toxicity, stability and mutagenicity (Ke et al., 2018; Su et al., 2018). Microbial degradation known as environmental friendly and cost effective, is an efficient method in removal of phenol (Acikgoz and Ozcan, 2016; Muñoz Sierra et al., 2018). To date, a large number of phenol-degrading bacteria affiliated with *Pseudomonas*, *Staphylococcus*, *Acinetobacter*, *Halomonas*, and *Bacillus*, etc., have been isolated from non-extreme environments, such as neutral pH environments (Senthilvelan et al., 2014; Van Dexter and Boopathy, 2018; Su et al., 2019b). Few studies focused on investigating phenol-degrading bacteria in acidic environments. However, neutrophilic microorganisms exhibited low performance for phenol degradation under acidic conditions, because the activity and stability of the phenol degrading enzymes were significantly susceptible to low pH (Huang et al., 2016; Bera et al., 2017). Thus, investigations on potential functional bacterial population in acidic environments are necessary to explore suitable communities for bioremediation of pollutant-contaminated environments with low pH.

Moreover, industrial phenolic wastewaters generated from coke production and chemical compounds production, contained high concentrations of salts (Ren et al., 2018; Su et al., 2018). High salinity limited microbial degradation of phenol and resulted in the biodegradation processes ineffective (Su et al., 2019b). High salinity (>10 g/L) could cause cell plasmolysis and death of microorganisms, which were attributed to osmotic pressure across the cell membranes (Muñoz Sierra et al., 2018). Apart from high salinity, the presence of heavy metals can also inhibit microbial enzymatic activity, which deteriorate the performance of biological treatment of industrial phenolic wastewaters (Ontañon et al., 2017). Isolation and application of microorganisms with the capability of degrading phenol and tolerance of heavy metals as well as a wide range of environmental conditions, is a useful method for enhanced removal of phenol in extreme environments. For example, Fernández et al. (2017) reported that many isolates obtained from extreme environments could utilize phenol, *n*-hexadecane and methanol as sole carbon sources, and were tolerant to 1 mM of copper, chromium and cadmium ions. Similarly, Jiang et al. (2016a) found that a strain *Debaryomyces* sp. JS4 was promising in treating some phenolic wastewater containing heavy metals and high salinity. It is reasonably expected that strains isolated from extreme environments are potential degraders under extreme conditions.

Acid mine drainage (AMD) is a typically extreme environment with low pH, high salinity, toxic heavy metals and metalloids, which has shaped unique AMD microbial communities (Akcil and Koldas, 2006). Microorganisms survived in the AMD environment have evolved various mechanisms to tolerate low pH, high salinity, toxic heavy metals, and organic contaminants (Huang et al., 2016). Recently, culture-independent methods, in particular those using high-throughput sequencing, deepen the understanding of the microbial diversity and community functions of AMD, and provide insight into the potential degraders in AMD environments (Huang et al., 2016; Aguinaga et al., 2018). However, to elucidate the function and characterization of microorganisms, a great need has existed for isolating pure strains (Su et al., 2019b, c). Therefore, it is meaningful to explore phenol-degrading strains with pH, salinity and heavy metal tolerances from AMD environments.

However, to the best of our knowledge, few studies have characterized phenol degradation potential of bacteria isolated from AMD environments. Therefore, in this study, a bacterial strain *Cobetia* sp. SASS1 was isolated from an AMD site, and comparatively analyzed with the most closely related species in genus *Cobetia*. Meanwhile, the effects of initial pH, salinity (in terms of NaCl) and heavy metals on phenol-degrading capability of SASS1 were evaluated by serial batch tests. Moreover, under optimal pH and salinity conditions, phenol degradation potential of SASS1 was assessed at various phenol concentrations. Importantly, together with chemical oxygen demand (COD) removal, metabolites identification and enzymes activities analysis, the mineralization pathway of phenol by SASS1 was proposed. This study provides a new insight into the degradation potential of indigenous microorganisms in AMD environments, which was important to explore high-efficient degraders under extreme environmental conditions.

## Materials and Methods

### Enrichment and Cultivation

Original wastewater sample was collected from natural pyrite site, which is very close to a coke factory area in Lishui city (Zhejiang, China). The wastewater sample was inoculated (10%, v/v) into mineral salt medium (MSM) containing (per liter): 2 g KH_2_PO_4_, 1.3 g Na_2_HPO_4_, 0.1 g (NH_4_)_2_SO_4_, and 0.01 g FeCl_3_, in which 100 mg/L phenol was added as the sole carbon and energy source, and adjusted pH to 2.8. The culture was incubated at 30°C on a rotary shaker at 130 rpm for 6 days. Then the culture was successively acclimated in the fresh MSM with increasing concentrations of phenol, ranging from 100 to 500 mg/L. Subsequently, the culture was diluted in 10-fold series, and 0.2 mL of serial dilutions (10^–2^ to 10^–8^) was spread on MSM agar plates with phenol as the carbon source. The purified colonies were then transferred to liquid MSM and the isolate SASS1 with the highest phenol degradation performance was selected for further experiments.

### Morphological, Cultural and Physiological Characteristics of the Strain SASS1

The strain SASS1 was inoculated (5%, v/v) in BSYG medium (pH adjusted to 2.8) containing (per liter): 2.0 g (NH_4_)_2_SO_4_, 0.1 g KCl, 0.25 g K_2_HPO_4_, 0.25 g MgSO_4_⋅7H_2_O, 0.01 g Ca(NO_3_)_2_, 1.0 g glucose and 0.1 g yeast extra. Morphological characteristics of SASS1 was observed using electron microscope with Gram staining and scanning electron microscopy (SEM, Hitachi S-4800). Moreover, the utilization of 71 different carbon sources and resistance to 23 inhibitory compounds were determined using GenIII Microplates Biolog^®^ Phenotype MicroArray (Biolog Inc.). Briefly, the pure colony was suspended in IF-A inoculating fluid (Biolog Inc.) and adjusted to 98% transmittance. Hundred μL of cell suspension was inoculated into the wells which contain the tetrazolium dye chemistry and appropriate nutrients. Formation of an irreversible purple formazan was observed to identify the utilization of different carbon sources by SASS1 (Bochner, 1989).

The batch experiments regarding the effects of temperature, pH and NaCl on SASS1 growth were performed in BSYG medium at different levels. Temperature-dependent growth was performed at various temperature including 5, 10, 15, 20, 25, 30, 35, and 40°C. For investigation of the optimal pH, bacterial cells were cultured at optimized temperature with various initial pH 2.0–10.0 in the step of 1.0. Meanwhile, at optimized pH and temperature, NaCl-tolerant concentration was tested at varied concentrations of NaCl (0, 15, 30, 45, 60, 75, 90, 105, and 120 g/L). All the experiments were performed in triplicate on a rotary shaker at 130 rpm for 48 h, and then cell growth was monitored by measuring the optical density at 600 nm (OD_600_) with a spectrophotometer (TU-1810, Purkinje, China).

### 16S rRNA Gene Analysis of the Strain SASS1

Total genomic DNA of the strain SASS1 was extracted using the EZ-10 spin column genomic DNA miniprep kit (Bio Basic Inc., Toronto, ON, Canada). PCR amplification was performed with a pair of universal bacterial primers 8F (5′-AGAGTTTGATCCTGGCTCAG-3′) and 1541R (5′-AAG GAGGTGATCCAGCCGCA-3′) (Löffler et al., 2000). PCR was conducted using 25 μL reaction mixture containing 0.5 μL template DNA, 12.5 μL 2 × Taq Master Mix, 10 μL RNase-free water and 1 μL 10 μM of each primer. The reaction started with initial denaturation at 94°C for 5 min, followed by 30 cycles of denaturation at 94°C for 30 s, annealing at 56°C for 30 s, extension at 72°C for 1 min and a final extension at 72°C for 5 min. Phylogenetic analyses based on the neighbor-joining algorithm was performed using the MEGA7 program (Kumar et al., 2016). The sequence was submitted to NCBI GenBank under the accession number of MH087429.

### Effects of pH, Salinity and Heavy Metals on Phenol Degradation

The cell suspension (OD_600_ = 1.1) was inoculated (5%, v/v) into MSM with 500 mg/L phenol as the sole carbon source. The effect of pH was investigated with various initial pH values ranging from 3.0 to 9.0 at interval of 1.0. Meanwhile, at the optimal pH, the salt tolerance of SASS1 was tested at NaCl concentrations of 0, 10, 20, 40, 60, 80, 100, and 120 g/L. The cell growth and residual concentration of phenol were examined every 10 h. The residual phenol was measured using 4-aminoantipyrine spectrophotometric method (Fiamegos et al., 2002). Phenol degradation efficiency was calculated as [(C_0_- C_1_)/C_0_] × 100%, where C_0_ and C_1_ are the initial and residual concentration of phenol, respectively (Su et al., 2018). All the experiments were performed in triplicate, and the data were expressed as means and standard deviation (SD).

Moreover, at the optimal pH and salinity, the tolerance of the strain SASS1 to heavy metals was assessed. SASS1 was inoculated (5%, v/v) into MSM containing 500 mg/L phenol with 0.2 mM of copper, zinc, cobalt, nickel and manganese, respectively. These heavy metal ion solutions were prepared by dilution of CuSO_4_⋅5H_2_O, ZnSO_4_⋅7H_2_O, CoCl_2_⋅6H_2_O, NiCl_2_⋅6H_2_O and MnCl_2_⋅4H_2_O, respectively. In addition, cultures without heavy metal addition were incubated in parallel as negative controls. All the cultures were incubated on a rotary shaker (130 rpm) in the dark for 40 h at 35°C. The residual concentration of phenol in each culture was measured at 10 h interval, and all the experiments were performed in triplicate.

### Phenol-Degrading Capability of the Strain SASS1

At optimal pH and salinity, the cell suspension (OD_600_ = 1.1) was inoculated (5%, v/v) into MSM at various phenol concentrations including 500, 700, 900, 1100, 1300, and 1500 mg/L, respectively. Each culture was incubated on a rotary shaker at 130 rpm and 35°C for 80 h. The cell growth and residual concentration of phenol in each culture were measured every 20 h. Meanwhile, at initial phenol concentration of 900 mg/L, the residual concentration of phenol, cell growth and COD were measured every 12 h. The COD concentration was determined according to the standard method (APHA, 1998). All the experiments were carried out in triplicates.

### Phenol Degradation Pathway by the Strain SASS1

The strain SASS1 was incubated under the same conditions (130 rpm, 35°C) in MSM with 900 mg/L phenol at optimal pH and salinity. Each culture (30 mL) was grown in 50 mL Erlenmeyer flask sealed with Teflon-lined rubbers stopper. Detection and identification of metabolites were performed by gas chromatography-mass spectrometry (GC–MS) (Agilent, Germany) (Su et al., 2019a). Briefly, the culture supernatants were collected at 20 h and 40 h by centrifugation, and then acidified to pH below 2.0 with 6 M HCl. The samples were then extracted three times with 20 mL of ethyl acetate. The three fractions of ethyl acetate extracts were mixed, dried through anhydrous Na_2_SO_4_, and then concentrated to near dryness by rotary evaporator. Then 0.1 mL *N,O*-bis(trimethylsilyl) acetamide and equal volume of *n*-hexane were added to the derivation and heated under a water bath at 60°C for 30 min (Nie et al., 2016). The resulting solution were analyzed by GC–MS (Hoyos-Hernandez et al., 2014). Highly pure helium was used as the carrier gas with a flow rate of 1.0 mL/min. The initial oven temperature of 60°C was maintained for 1 min, raised to 160°C at a rate of 5°C/min, and then raised to 200°C at a rate of 5°C/min (held for 5 min), finally raised to 280°C at a rate of 15°C/min (held for 5 min). The detector and injector temperatures were 230 and 280°C, respectively. MS was operated in electron-impact (EI) mode with a scan range of m/z 30–450.

Based on retention time and mass spectrum analysis, as well as comparison with national institute of standards and technology (NIST) database (Gaithersburg, MD, United States)^1^, the chromatographic peaks were identified (Lu et al., 2018). In addition, to further verify the degradation metabolites, the benzoic acid concentration after 40 h of phenol degradation was measured by HPLC (Ultimate 3000, Dionex) according to the method described by Huang et al. (2015). Meanwhile, the activities of phenol hydroxylase (EC 1.14.13.7) and catechol 1,2-dioxygenase (EC 1.13.11.1) were detected by UV-vis spectrophotometer (TU-1810, Purkinje, China) at wavelengths of 340 nm and 260 nm, respectively (Jiang et al., 2007). In brief, the above culture supernatants collected at 20 and 40 h were washed twice with 0.1M phosphate sodium buffer (pH 7.2), and then the resuspension was disrupted by sonication for 5 min. After centrifugation, the supernatant was used for assays of enzymes and total protein. Total protein concentration was determined using Bradford quantification kit (Sangon, Shanghai, China). The specific activities of enzymes were expressed as units (U) per milligram of total cell protein (Jiang et al., 2007).

## Results

### The Characteristics and Identification of SASS1

The strain SASS1 was milky white, moist, and Gram-negative (Supplementary Figure S1). It was able to grow at low temperature and get the best growth at 35°C (Supplementary Figure S2A). At optimized temperature of 35°C, the cell growth was observed to occur at pH ranging from 2.0 to 10.0 (optimum 4.0), but the growth rate was retarded at pH value of 2.0 or higher than 9.0 (Supplementary Figure S2B). For salinity tolerance, the cell growth was decreased with increasing concentrations of NaCl, and the maximum NaCl-tolerance concentration was 90 g/L (Supplementary Figure S2C). The results of cultural characteristics demonstrated that the growth (OD_600_ > 0.2) of SASS1 occurs at wide ranges of temperature (10–35°C), pH (3.0–10.0) and salinity (0–90 g/L). Meanwhile, the strain SASS can use 32 out of 71 tested carbon sources (Supplementary Table S1), and is capable of resistance to eight inhibitory chemical compounds including aztreonam, lithium chloride, niaproof 4, sodium butyrate, sodium chloride (1, 4, and 8%, w/v), sodium lactate (1%), tetrazolium blue and vancomycin (Supplementary Table S2).

**FIGURE 1:**
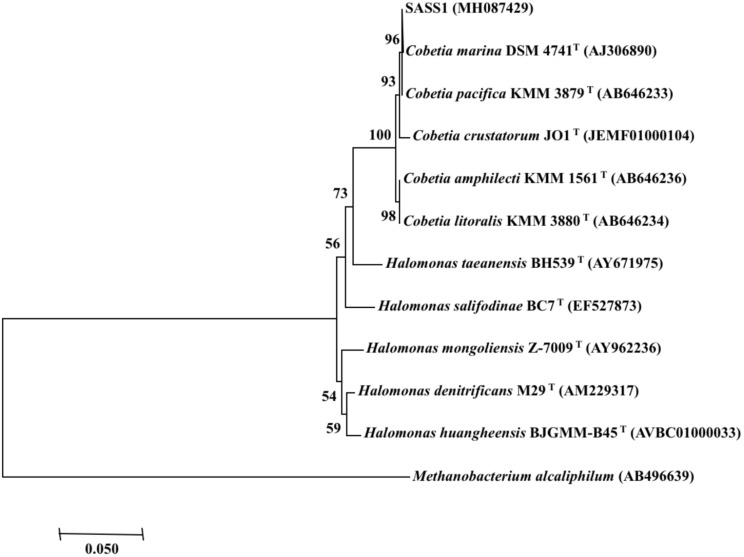
Neighbour-joining phylogenetic tree of bacterial 16S rRNA gene sequences, including the strain SASS1 and 10 of their most similar sequences from the EzBioCloud database. Numbers above branches indicate bootstrap support (1000 replicates). *Methanobacterium alcaliphilum* was used as an outgroup.

Phylogenetic analysis of 16S rRNA gene of SASS1 was shown in Figure 1. The strain shared the highest 16S rRNA gene similarities (99.2–100%) with *Cobetia marina* DSM 4741^T^, *Cobetia pacifica* KMM 3879^T^, *Cobetia amphilecti* KMM 1561^T^, *Cobetia litoralis* KMM 3880^T^, and *Cobetia crustatorum* JO1^T^. The physiological characteristics of SASS1 compared with the five closely related type strains were presented in Table 1. The results suggested that the strain *Cobetia* sp. SASS1 presented different cultural and physiological characteristics in comparison with the five most closely related species.

**TABLE 1:** Differential characteristics of the strain SASS1 and the most closely related species of genus *Cobetia.*

**Characteristic**	**SASS1**	***Cobetia marina* DSM 4741^T^**	***Cobetia pacifica* KMM 3879^T^**	***Cobetia amphilecti* KMM 1561^T^**	***Cobetia litoralis* KMM 3880^T^**	***Cobetia crustatorum* JO1^T^**
Motile	+	–	+	+	+	+
Temperature range (°C)	10–35	10–42	4–42	4–42	4–42	4–30
Optimal temperature(°C)	35	37	35–38	37	35–37	25
pH range	2–10	5–10	4.0–11.0	4.5–10.5	5.0–10.5	5–10
Optimal pH	3.0–8.0	7.5	7.5–8.5	6.5–8.5	7.5–8.5	5.0–6.0
NaCl range (g/L)	0–120	5–200	0–200	0–200	0–200	35–115
Optimal NaCl (g/L)	0–45	50–100	50–60	50	50–60	65
Growth on:						
D-glucose	+	+	+	+	+	+
Maltose	+	–	(+)	+	(+)	+
D-mannose	+	+	–	–	–	+
D-fructose	+	–	–	(+)	–	+
D-galactose	+	+	–	+	+	+
D-mannitol	+	–	–	–	+	+
Cellobiose	+	+	+	+	(+)	+
D-lactose	+	+	–	+	(+)	–
*myo-*Inositol	–	+	–	+	–	–
Sucrose	+	–	+	+	–	+
Salicin	–	+	+	+	(+)	–
Melibiose	–	+	+	+	–	–
*N*-acetylglucosamine	+	+	+	+	–	–
Raffinose	–	ND	–	–	–	–
L-rhamnose	–	+	+	+	–	
L-fucose	–	+	–	+	–	–
D-sorbitol	+	+	+	–	–	–
Propionic acid	+	–	+	–	–	–
L-histidine	–	–	+	+	+	+
L-alanine	–	–	ND	+	ND	+
L-proline	–	–	ND	+	ND	+
L-serine	+	–	ND	+	ND	+
Glycerol	–	–	–	–	–	+
Gelatin	+	–	–	–	–	–
Vancomycin	+	–	+	+	–	ND
Rifampicin	–	–	(+)	–	–	ND
Lincomycin	–	ND	+	+	–	ND
Isolation source	Acid mine drainage	Coastal sea sample	Marine sediments	Internal tissue of sponge	Marine environments	Salt-fermented food
References	This study	Arahal et al., 2002	Romanenko et al., 2013	Romanenko et al., 2013	Romanenko et al., 2013	Min-Soo, 2010

*“+”, positive; “–”, negative; “(+)”, weak reaction; “ND”, Not determined.*

### Effects of pH and Salinity on Phenol Degradation

**FIGURE 2:**
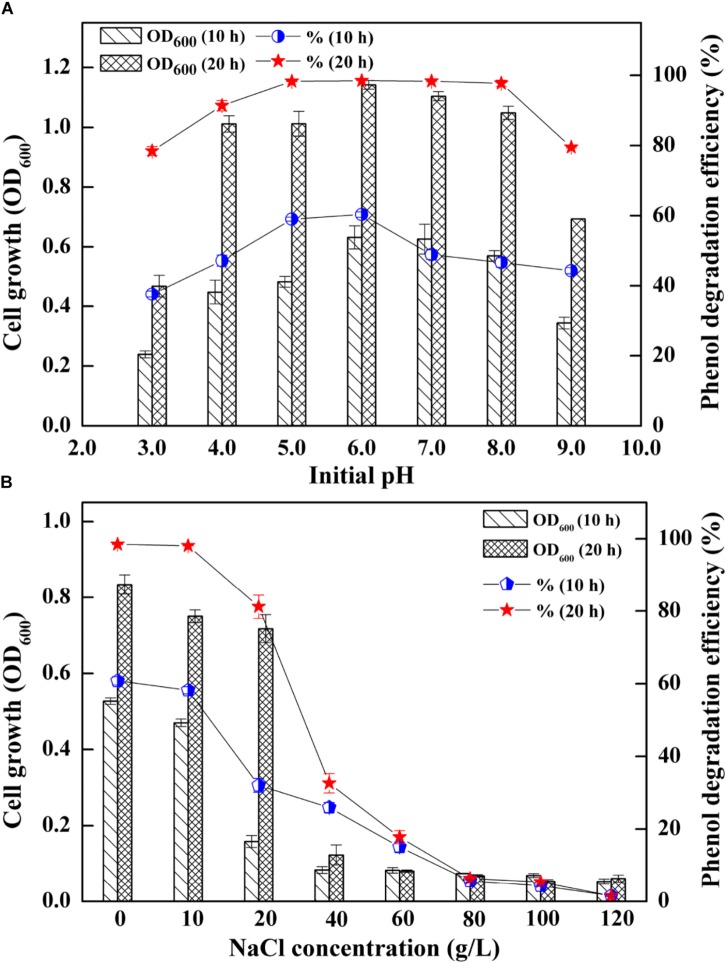
Effects of different initial pH **(A)** and salinities **(B)** on the cell growth and phenol degradation efficiency.

The AMD sites are characterized by low pH and a wide range of salinities (Kuang et al., 2013), which enabled the strain SASS1 more tolerance of pH and salinities variations. Thus, SASS1 may have potentials for phenol degradation under different pH and salinity stresses. The effects of different pH and salinities on the cell growth and phenol degradation efficiency of SASS1 were shown in Figure 2. It was observed that SASS1 could degrade the phenol almost completely within 20 h when the initial pH was 5.0, 6.0 and 7.0 (Figure 2A). The OD_600_ underwent an increasing trend, reaching its maximum value of 1.14 at pH 6.0, and then decreased to 0.69 at pH 9.0. Similar to the trend of variations in cell growth, the degradation of phenol was decreased from 98.47 to 79.50% with elevated pH from 6.0 to 9.0. The results suggested that SASS1 could efficiently degrade phenol in the wide range of pH 4.0–8.0. At the optimal pH of 6.0, the phenol degradation and cell growth under various salinity conditions were shown in Figure 2B. It could be observed from Figure 2B that phenol degradation efficiency reached almost 100% within 20 h at NaCl concentration of 10 g/L. At 20 g/L NaCl, phenol degradation efficiency increased from 32.0 to 81.2% with incubation time increasing from 10 to 20 h. When the concentration of NaCl was higher than 20 g/L, phenol degradation efficiency decreased significantly with increasing NaCl concentration. At NaCl concentration of 60 g/L, the degradation efficiency was only 17.8% after 20 h. Similar to the trend of phenol degradation efficiency, the value of OD_600_ was decreased with the elevated NaCl concentrations. When the NaCl concentration increasing from 20 to 60 g/L, the OD_600_ value decreased from 0.71 to 0.08. The results demonstrated that phenol degradation was significantly inhibited when NaCl concentration was higher than 20 g/L, and little growth was observed when NaCl concentration were higher than 60 g/L. This suggested that phenol degradation of SASS1 was significantly affected by high NaCl concentration (>20 g/L). Therefore, the optimal pH and salinity for phenol degradation by SASS1 were 6.0 and 10 g/L, respectively.

### Tolerance of Heavy Metals

**FIGURE 3:**
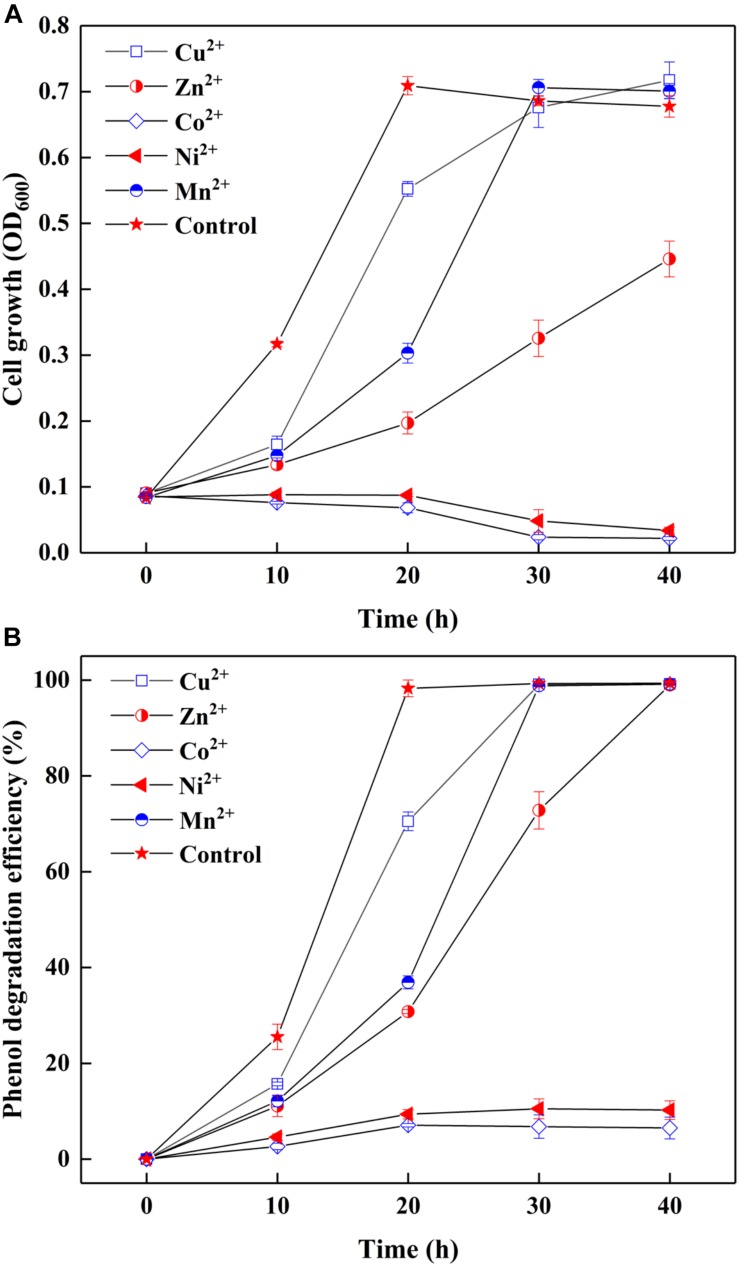
Effect of heavy metals on cell growth **(A)** and phenol degradation **(B)** at 500 mg/L phenol and 10 g/L NaCl.

Since most of AMD sites are heavy metal-rich (Aguinaga et al., 2018), the survived strain SASS1 under such extreme environments could also be tolerant of heavy metals during phenol degradation. The effect of heavy metals on cell growth and phenol degradation were investigated at the optimal pH and salinity. It could be seen from Figure 3A, although the addition of Cu^2+^ or Mn^2+^ retarded the growth of SASS1, the OD_600_ values could achieve almost the same values as control group after 30 h. However, the OD_600_ value of samples containing heavy metal of Zn^2+^, Co^2+^, or Ni^2+^ decreased remarkably when comparing with the control group. For example, no obvious growth could be observed in media with Co^2+^ or Ni^2+^ after 30 h. Moreover, as depicted in Figure 3B, in control group, phenol could be completely degraded within 20 h which was shorter than the time needed for phenol degradation with heavy metal addition. For addition of Cu^2+^ or Mn^2+^, almost complete degradation of phenol was observed within 30 h, whereas 40 h was needed for addition of Zn^2+^. Serious inhibitions of phenol biodegradation were observed when Co^2+^ or Ni^2+^ was added to cultures. From 10 to 40 h, phenol degradation efficiencies increased from 2.7 to 6.5% with addition of Co^2+^, and 4.7 to 10.2% with addition of Ni^2+^. The results indicated that SASS1 could maintain the metabolic activity for treatment of some kinds of phenol-laden saline wastewater containing heavy metals.

### Phenol-Degrading Capability of the Strain SASS1 Under Salinity Condition

**FIGURE 4:**
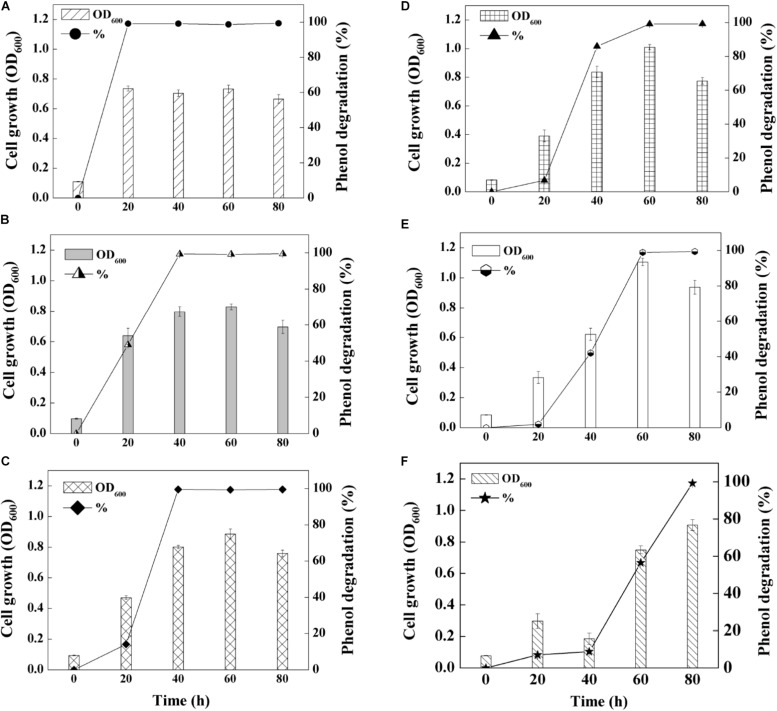
Phenol-degrading capabilities of the strain SASS1 at various phenol concentrations of 500 mg/L **(A)**, 700 mg/L **(B)**, 900 mg/L **(C)**, 1100 mg/L **(D)**, 1300 mg/L **(E)** and 1500 mg/L **(F)** with 10 g/L NaCl.

On basis of the optimal pH and salinity assessment, as well as heavy metal tolerance evaluation, phenol degradation capability of SASS1 was investigated under various phenol concentrations. As shown in Figure 4, the growth of SASS1 was inhibited by phenol at the beginning, but achieved the maximum value of 1.11 after 60 h at 1300 mg/L phenol. The lag phase lengthened from 20 to 40 h with phenol concentration increasing from 1300 to 1500 mg/L, which demonstrated that the growth of SASS1 was greatly affected by phenol concentrations higher than 1300 mg/L. As similar trend was observed for phenol degradation. With increasing phenol concentration from 900 to 1500 mg/L, the phenol degradation efficiency decreased from 99.5 to 8.8% after 40 h. Almost complete degradation of 1300 and 1500 mg/L phenol could be observed within 60 and 80 h, respectively. The results suggested that SASS1 could be quick adaptation of high phenol concentration, and showed excellent performance in phenol degradation.

**FIGURE 5:**
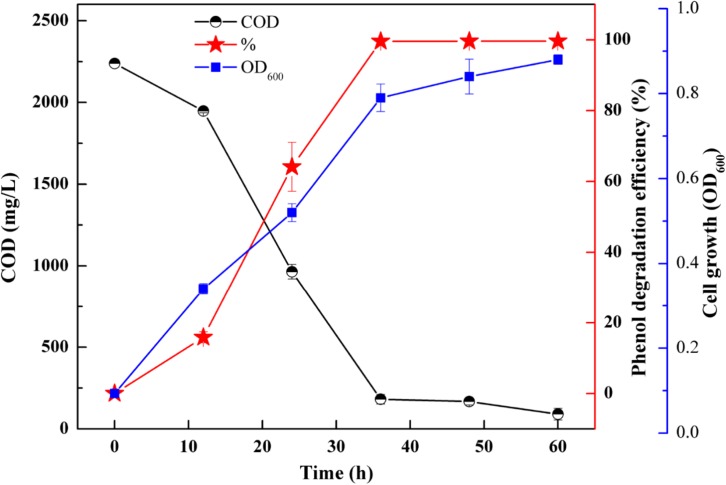
COD removal, phenol degradation and cell growth at 900 mg/L phenol and 10 g/L NaCl.

In order to understand the mineralization ability of SASS1 for phenol degradation, COD removal following phenol degradation was performed at 900 mg/L phenol with 10 g/L NaCl. Figure 5 represents the variations of COD concentration, phenol degradation efficiency and cell growth with increasing incubation time from 0 to 60 h. After 36 h, SASS1 demonstrated 100% phenol degradation efficiency and OD_600_ value of 0.79 which was close to the maximum OD_600_ value (0.88), while COD decreased from the initial concentration of 2239 to 181.6 mg/L. The COD continued to decrease after complete phenol degradation, and the value of 90.3 mg/L was achieved within 60 h.

### Proposed Pathway for Phenol Degradation by the Strain SASS1

**FIGURE 6:**
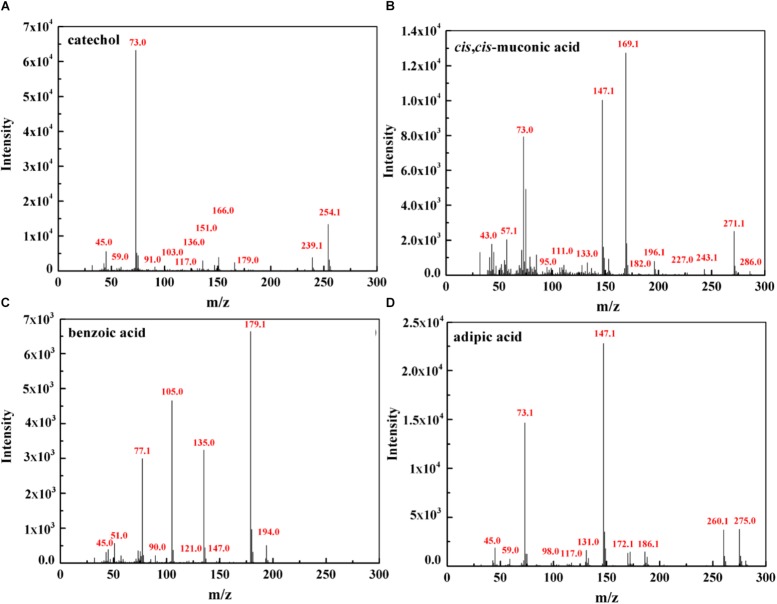
Mass spectra of the metabolites identified during phenol degradation by the strain SASS1. Catechol **(A)** and *cis*, *cis*- muconate **(B)** detected at 20 h; benzoic acid **(C)** and adipic acid **(D)** detected at 40 h.

**FIGURE 7:**
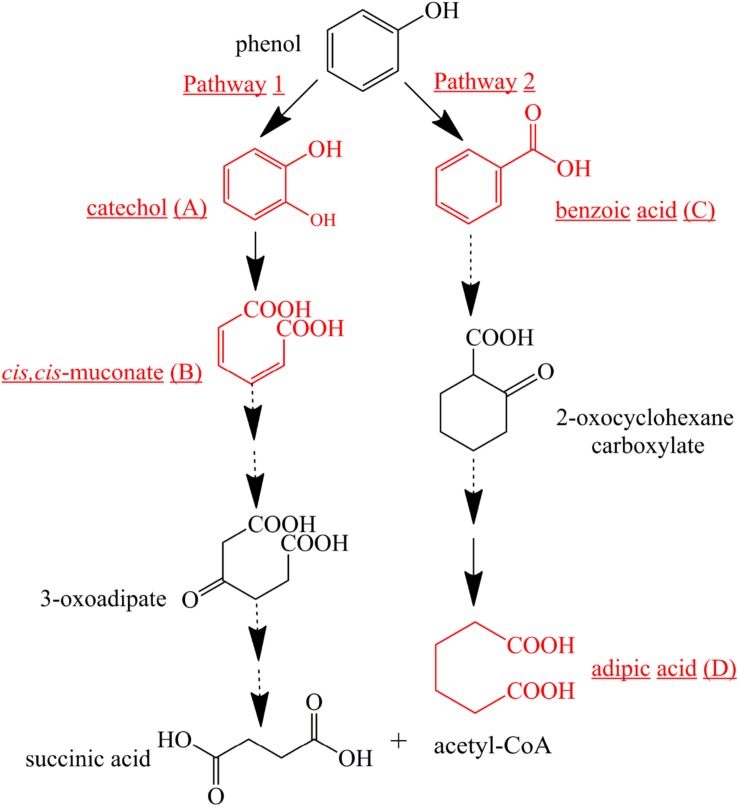
Proposed pathways for phenol degradation by the strain SASS1. The solid line shows the observed degradation pathway, and the dashed line shows the predicted pathways from previous studies (Hoyos-Hernandez et al., 2014; Nesvera et al., 2015).

To further investigate phenol mineralization by SASS1, degradation metabolites after 20 and 40 h incubation were monitored by GC-MS analysis at initial concentration of 900 mg/L. Four obvious peaks relevant to phenol metabolism are shown in Supplementary Table S3, and the mass spectra of the four metabolites catechol (*m/z* 254), *cis*, *cis*-muconate (*m/z* 286), benzoic acid (*m/z* 194) and adipic acid (*m/z* 290), are illustrated in Figure 6. Residual phenol with retention time (Rt) of 8.397 min was detected after 20 h incubation, which was consistent with the results of 36 h for complete degradation of 900 mg/L phenol. Peaks which represented metabolites catechol, *cis*, *cis*-muconate, benzoic acid and adipic acid, were detected at retention time of 15.438, 21.635, 13.461, and 11.737 min, respectively. At 20 h, the peaks of catechol and *cis*, *cis*-muconate were detected, while at 40 h, the peak of catechol disappeared, and the intensity of peak of *cis*, *cis*-muconate become higher. Meanwhile, the peaks of benzoic acid and adipic acid were detected. In order to further verify the results of GC-MS analysis, the activities of phenol hydroxylase and catechol 1,2-dioxygenase were investigated. At 20 h, the presence of absorbance at 340 and 260 nm indicated the occurrence of catechol and *cis*, *cis*- muconate, respectively, and the specific activities of the phenol hydroxylase and catechol 1,2-dioxygenase were 0.621 ± 0.082 and 0.273 ± 0.019 U/mg, respectively. At 40 h, the absence of absorbance at 340 nm was consistent with the absence of catechol by GC-MS analysis, and the specific activity of the catechol 1,2-dioxygenase was 0.741 ± 0.063 U/mg. Meanwhile, the concentration of benzoic acid detected at 40 h was 10.3 mg/L. These findings further verified the metabolites detected by GC-MS analysis. Two possible degradation pathways for phenol degradation by the strain SASS1 were presented in Figure 7. For the pathway 1, phenol was firstly transferred to catechol by phenol hydroxylase, then the ring of catechol was oxidized by the catalysis of catechol 1,2-dioxygenase to form *cis*, *cis*-muconate, following *ortho*-pathway. For the pathway 2, phenol was converted to benzoic acid and then to adipic acid. Based on the intensity of peaks and benzoic acid concentration detected, it could be deduced that benzoic acid pathway may play a minor role in phenol degradation. Finally, some intermediates could be further oxidized into CO_2_ and H_2_O, which were supported by the COD removal shown in Figure 5. In light of these results, it is clear that the strain SASS1 possessed the ability to mineralize phenol.

## Discussion

The strain SASS1 isolated from AMD environment possessed the distinctive cultural and physiological characteristics in comparison with the most closely related species, which suggested that SASS1 may represent a novel species of genus *Cobetia*. Further investigations of chemotaxonomic and molecular characteristics in comparison with the five species are needed in the future. The optimum pH of 6.0 for phenol degradation by SASS1 was different from the optimum pH of 4.0 for cell growth in BSYG medium, which may be attributed to the higher enzymes activities involved in phenol degradation at pH 6.0 (Acikgoz and Ozcan, 2016). Notably, the strain SASS1 exhibited high phenol degradation efficiency with the initial pH values ranging from 3.0 to 9.0, which demonstrated the advantage of SASS1 in degrading phenol in a wide pH range. In the past two decades, most of isolated phenol-degrading microorganisms were neutral, few could maintain metabolic activities in a wide range of pH (Jiang et al., 2015; Su et al., 2019c). Bera et al. (2017) found that most microbial enzymes tended to lose the catalytic activity under extreme pH conditions.

Meanwhile, the effects of salinity on cell growth and phenol degradation of SASS1 indicated that high NaCl concentrations (>20 g/L) were detrimental to cell growth and deteriorated phenol degradation. This phenomenon was consistent with the report that high salt concentration led to hypertonic osmotic imbalance and loss of metabolic activity of degraders (Jiang et al., 2015; Su et al., 2019b). However, at initial phenol concentration of 500 mg/L, the strain SASS1 could degrade more than 25% of phenol within 10 h at 40 g/L NaCl, which demonstrated the good performance of SASS1 in degradation of phenol at high salinity. Although previous studies have demonstrated the metabolic activity of phenol-degrading bacterial members in the family *Halomonadaceae* under high salinity (Alva and Peyton, 2003; Jiang et al., 2015, 2016b), there are few studies reporting phenol-degrading capability of bacterial members in genus *Cobetia* under conditions of acidity or high salinity. It is worth noting that the higher phenol degradation efficiency of SASS1 was revealed when comparing with the phenol degradation capacities of reported phenol-degrading bacterial strains in literature (Supplementary Table S4). Meanwhile, under high salinity conditions, SASS1 also possessed better performance in phenol degradation than that of *Cobetia marina* EBR04 reported by Kobayashi et al. (2012). For example, after 20 h, the strain SASS1 could degrade 81% phenol at initial concentration of 500 mg/L with NaCl concentration of 20 g/L, while *Cobetia marina* EBR04 could degrade 80% phenol at initial phenol concentration of 100 mg/L with NaCl concentration of 15 g/L (Kobayashi et al., 2012). Therefore, it could be obtained that the *Cobetia* sp. SASS1 can degrade high concentration of phenol under wide pH and high salinity conditions.

Moreover, the strain SASS1 has the ability to tolerate heavy metals, which can further maintain superior performance in phenol degradation under heavy metal stress. As well known, many industrial plants generated phenolic wastewater with heavy metals, which resulted in the bad performance of biological wastewater treatment. Thus, isolation of phenol-degrading strains with the ability of heavy metal tolerance is important for enhancing biological treatment of complex phenolic wastewater (Jiang et al., 2016a). Wasi et al. (2011) found that a bacterial strain *Pseudomonas fluorescens* SM1 was capable of degrading phenol and surviving in heavy-metal contaminated environment. With regard to the genus *Cobetia*, although several studies have reported their resistance to metal ions (Ivanova et al., 2005), few studies have focused on the effects of heavy metals on phenol biodegradation. The heavy metal resistant ability of the phenol-degrading strain SASS1 was useful for biological treatment of phenolic wastewater with heavy metals, such as refinery wastewater (El-Naas et al., 2009; Jiang et al., 2016a).

In addition, the potential of phenol mineralization of the strain SASS1 was uncovered based on biodegradation metabolites and enzyme activities analysis. The COD removal after complete phenol degradation was consistent with the study by Afzal et al. (2007) which showed a longer time for COD removal compared with phenol degradation by *Pseudomonas pseudomallei*. Notably, the results are better as compared to the report that 2032 mg/L COD was reduced to 269 mg/L after 40 h incubation. Although pathways for aerobic phenol degradation have been extensively investigated (Nesvera et al., 2015; Zhou et al., 2016), there is no report elucidating the phenol degradation pathway by bacterial members in the genus *Cobetia*. In general, under aerobic conditions, phenol firstly converted to catechol by phenol hydroxylase, then catalyzed by either catechol 1,2-dioxygenase (C12O) to form *cis*, *cis*-muconate via *ortho*-pathway (Banerjee and Ghoshal, 2010), or catechol 2,3-dioxygenase (C23O) to form 2-hydroxymuconic semialdehyde (2-HMS) via *meta*-pathway (Tuan et al., 2011). In this study, SASS1 was assigned to both *ortho*-cleavage pathway and benzoic acid pathway, and converted phenol to non-toxic intermediates of succinic acid, acetyl-CoA and adipic acid. The metabolites of muconolactone, 3-oxoadipate enol-lactone, 3-oxoadipate or succinic acid were not detected, which may be attributed to that these metabolites are too transient to be detectable (Van Dexter and Boopathy, 2018). Interestingly, several studies have shown that aerobic phenol degradation via *ortho*- or *meta*- cleavage pathway, and not through the benzoic acid pathway which was generally existed under anaerobic conditions (Leven et al., 2006; Senthilvelan et al., 2014; Zhou et al., 2016). For example, Hoyos-Hernandez et al. (2014) found that in the anaerobic digestion of municipal solid waste, phenol was degraded via benzoic acid pathway. Levén and Schnürer (2005) reported that culture with mesophilic community converted phenol to benzoic acid at 37°C under anaerobic conditions. However, under aerobic conditions, similar results were obtained by Nie et al. (2016) who found that *P*-hydroxybenzoic acid was detected during phenol biodegradation. The benzoic acid pathway appeared during phenol degradation by SASS1 may be attributed to the lack of oxygen in the culture system. Further work is needed to uncover the shifts in phenol degradation pathway in response to different oxygen concentrations. It is worth noting that the *ortho*-cleavage pathway was very important to mineralize phenol by SASS1, because incomplete mineralization generally appeared in *meta*-cleavage pathway (Arora and Bae, 2014). Therefore, the pH- and salt-tolerant strain *Cobetia* sp. SASS1 which was efficient for phenol mineralization, could have potential applications in treating phenol-laden saline wastewater with heavy metals. These findings broaden our insight into bioremediation of AMD sites contaminated with aromatic compounds and heavy metals, and highlight the role of the genus *Cobetia* in phenol degradation under stressful conditions.

Together, the pH- and salt-tolerant phenol-degrading strain *Cobetia* sp. SASS1 which presented different cultural and physiological characteristics with the most closely related species, was isolated from the AMD site. The strain can efficiently degrade phenol in wide ranges of pH (3.0–9.0) and NaCl concentration (0–40 g/L), as well as the existence of heavy metals. At 10 g/L NaCl, SASS1 was capable of complete degradation of 1300 mg/L phenol within 60 h, and 900 mg/L phenol within 36 h. Importantly, phenol mineralization by SASS1 was assigned to both catechol 1,2-dioxygenase activity of *ortho*-cleavage pathway and benzoic acid pathway. This study provides new insights into the potential of indigenous bacterial species in AMD for biological treatment of complex saline phenolic wastewater.

## Data Availability

All datasets generated for this study are included in the manuscript and/or the Supplementary Files.

## Author Contributions

XS, RM, and MZ conceived and designed the study. RM, MZ, LX, and YZ performed the experiments and analyzed the data. XS wrote the manuscript.

## Conflict of Interest Statement

The authors declare that the research was conducted in the absence of any commercial or financial relationships that could be construed as a potential conflict of interest.

## Footnotes

1 http://www.nist.gov/srd/mslist.htm
