# Supplementary material for: Characterization of a pH-Tolerant Strain Cobetia sp. SASS1 and Its Phenol Degradation Performance Under Salinity Condition
Source: Front Microbiol. 2019 Sep 4;10:2034. doi: 10.3389/fmicb.2019.02034 (PMC6737460; doi:10.3389/fmicb.2019.02034)
Supplement: Supplementary file 1 [file Data_Sheet_1.docx]

**Supplementary material**

**Characterization of a pH-tolerant strain *Cobetia* sp. SASS1 and its phenol degradation performance under salinity condition**

Rongwu Mei^1,#^, Meng Zhou^2,#^, Luning Xu^2^, Yu Zhang^1^, Xiaomei Su^2,^*

*^1^Environmental Science Research and Design Institute of Zhejiang Province, Hangzhou 310007, China*

*^2^College of Geography and Environmental Science, Zhejiang Normal University, Jinhua 321004, China*

*Corresponding author: Xiaomei Su

Address: Yingbin Road 688#, Jinhua, 321004, China

E-mail: [purple@zjnu.cn](mailto:purple@zjnu.cn)

^#^These authors have contributed equally to this work.

**Table S1** Carbon substrates found in the different microtitre plates from BIOLOG.

| Substrate | Activity | Substrate | Activity |
| --- | --- | --- | --- |
| α­D­Glucose  α­D­Lactose  β-Methyl-D-glucoside  3-Methyl glucose  D-Arabitol  D-Cellobiose  D-Fructose  D-Fucose  D-Galactose  D-Maltose  D-Mannitol  D-Mannose  D-Melibiose  D-Raffinose  D­Salicin  D-Sorbitol  D-Trehalose  D­Turanose  Gentiobiose  L-Fucose  L-Rhamnose  N-Acetyl-D-galactosamine  N-Acetyl-D-glucosamine  N-Acetyl-β­D-mannosamine  N-Acety Neuraminic Acid  Pectin  Stachyose  Sucrose  γ-Amino butyric acid  D-Aspartic Acid  D-Serine  Glycyl-L-proline  L-Alanine  L-Arginine  L-Aspartic acid  L-Glutamic acid | +  -  -  +  +  +  +  +  +  +  +  +  -  -  -  +  +  +  -  -  -  -  +  +  +  -  +  +  +  -  -  -  -  -  +  - | L-Histidine  L-Pyroglutamic acid  L-Serine  α-Hydroxy butyric acid  β-Hydroxy-D,L­butyric acid  α-Keto butyric acid  α-Keto glutaric acid  Acetic acid  Acetoacetic acid  Citric acid  D-Galacturonic acid  D-Gluconic acid  D-Glucuronic acid  D-Malic acid  D-Saccharic acid  Formic acid  L-Lactic acid  L-Malic acid  Mucic acid  p-Hydroxy phenylacetic acid  Propionic acid  Quinic acid  Dextrin  Tween 40  Glucuronamide  D-Lactic Acid Methyl Ester  L-Galactonic Acid Lactone  Methyl Pyruvate  Bromo-Succinic Acid  D­Fructose-6-phosphate  D­Glucose-6-phosphate  Gelatin  Glycerol  Inosine  myo-Inositol | -  +  +  -  -  +  +  +  +  -  +  -  +  -  -  -  -  -  -  -  +  -  -  -  +  -  -  -  -  +  -  +  -  -  - |

“+” positive result, “-” negative result.

**Table S2** Chemical sensitivity test of the strain SASS1.

| Characteristic | Chemical formula | Activity |
| --- | --- | --- |
| Resistance to inhibitory compound | | |
| Aztreonam  D-Serine  Fusidic acid  Guanidine HCl  Lincomycin  Lithium chloride  Minocycline  Nalidixic acid  Niaproof 4  Potassium tellurite  Rifamycin SV  Sodium bromate  Sodium butyrate  Sodium chloride (1%, w/v)  Sodium chloride (4%, w/v)  Sodium chloride (8%, w/v)  Sodium lactate (1%)  Tetrazolium blue  Tetrazolium violet  Troleandomycin  Vancomycin | C_13_H_17_N_5_O_8_S_2_  C_3_H_7_NO_3_  C_31_H_48_O_6_  CH_6_ClN_3_  C_18_H_35_ClN_2_O_6_S  LiCl  C_23_H_27_N_3_O_7_  C_12_H_12_N_2_O_3_  C_14_H_29_NaO_4_S  K_2_TeO_3_  C_37_H_46_NNaO_12_  NaBrO_3_  C_13_H_17_N_5_O_8_S_2_  NaCl  NaCl  NaCl  C_3_H_5_NaO_3_  C_40_H_34_N_8_O_2_  C_23_H_17_N_4_  C_41_H_67_NO_15_  C_66_H_76_C_l3_N_9_O_24_ | +  -  -  -  -  +  -  -  +  -  -  -  +  +  +  +  +  +  -  -  + |
| Growth at | | |
| pH 5  pH 6 |  | +  + |

“+” positive result, “-” negative result.

**Table S3** GC-MS analysis of main metabolites of phenol degradation by the strain SASS1 within 20 h and 40 h.

| Retention Time/min | Fragment ions observed and relative intensity | Chemical structure | Compound | Incubation time (h) |
| --- | --- | --- | --- | --- |
| 8.397 | 166 (M^+^, 29) 151 (100) 135 (5) 121 (3) 91 (7) 77 (7) 65 (3) 45 (3) |  | phenol | 20 |
| 15.438  (A) | 254 (M^+^, 21) 239 (6) 179 (1) 166 (4) 151 (6) 136 (5) 117 (1) 103 (1) 91 (2) 73 (100) 59 (2) 45 (9) |  | catechol | 20 |
| 21.635  (B) | 286 (M^+^, 2) 271 (20) 243 (3) 227 (1) 196 (6) 182 (1) 169 (100) 147 (79) 133 (6) 111 (5) 95 (4) 73 (62) 57 (16) 43 (14) |  | *cis*, *cis*- muconate | 20/40 |
| 13.461  (C) | 194 (M^+^, 8) 179 (100) 147 (1) 135 (49) 121 (1) 105 (70) 90 (3) 77 (45) 51 (9) 45 (6) |  | benzoic acid | 40 |
|  |  |  |  |  |
| 11.737  (D) | 275 (17) 260 (16) 186 (6) 172 (6) 147 (100) 131 (7) 117 (2) 98 (1) 73 (64) 59 (3) 45 (8) |  | adipic acid | 40 |

**Table S4** Comparison of the strain SASS1 in this study to previously phenol-degrading bacterial strains based on the capability to degrade phenol at high initial concentration and incubation conditions (pH and temperature).

| Bacterial strains | Phenol concentration  (mg/L) | NaCl (g/L) | Incubation conditions (pH/°C) | Degradation time (h) | References |
| --- | --- | --- | --- | --- | --- |
| *Kocuria* sp*.* TIBETAN4 | 471 | 0 | 8/25 | 72 | (Wu et al., 2018) |
| *Acinetobacter tandoii* | 280 | 0.10 | -/37 | 72 | (Van Dexter and Boopathy, 2018) |
| *Acinetobacter* sp. SA01 | 1000 | 0.5 | 7/30 | 60 | (Shahryari et al., 2018) |
| *Bacillus* sp. SAS19 | 1800 | 0 | 7/30 | 78 | (Ke et al., 2018) |
| *Modicisalibacter tunisiensis* LIT2 | 200 | 90 | -/28 | 120 | (Gomes et al., 2018) |
| *Pseudomonas* sp. PH7 | 500 | 0 | 7/30 | 48 | (Tian et al., 2017) |
| *Bacillus lichenformis* SL10 | 2000 | 0.1 | 7/37 | 96 | (Felshia et al., 2017) |
| *Sulfobacillus acidophilus* TPY | 100 | 0 | 1.8/45 | 40 | (Zhou et al., 2016) |
| *Chlorella* sp. | 500 | 0 | 7/28 | 168 | (Wang et al., 2016) |
| *Pseudomonas stutzeri* N2 | 400 | 0 | 7.3-7.5/30 | 30 | (Nie et al., 2016) |
| *Acinetobacter Calcoaceticus* PA | 800 | 0 | -/30 | >48 h | (Liu et al., 2016) |
| *Archaea* A235 | 100 | 200 | 7.35/37 | >350 h | (Acikgoz and Ozcan, 2016) |
| *Halomonas* sp. 4-5 | 500 | 30-100 | 7/30 | >68 h | (Lu et al., 2015) |
| *Pseudomonas putida* Tan-1 | 400 | 0 | 6/35 | 72 | (Senthilvelan et al., 2014) |
| *Cobetia marina* EBR04 | 100 | 15 | 7/30 | 40 | (Kobayashi et al., 2012) |
| *Cobetia* sp. SASS1 | 1500 | 10 | 6/30 | 80 | This study |
| *Cobetia* sp. SASS1 | 500 | 20 | 6/30 | 20 (> 80%)* | This study |

“*”means more than 80% of phenol was degraded within 20 h.


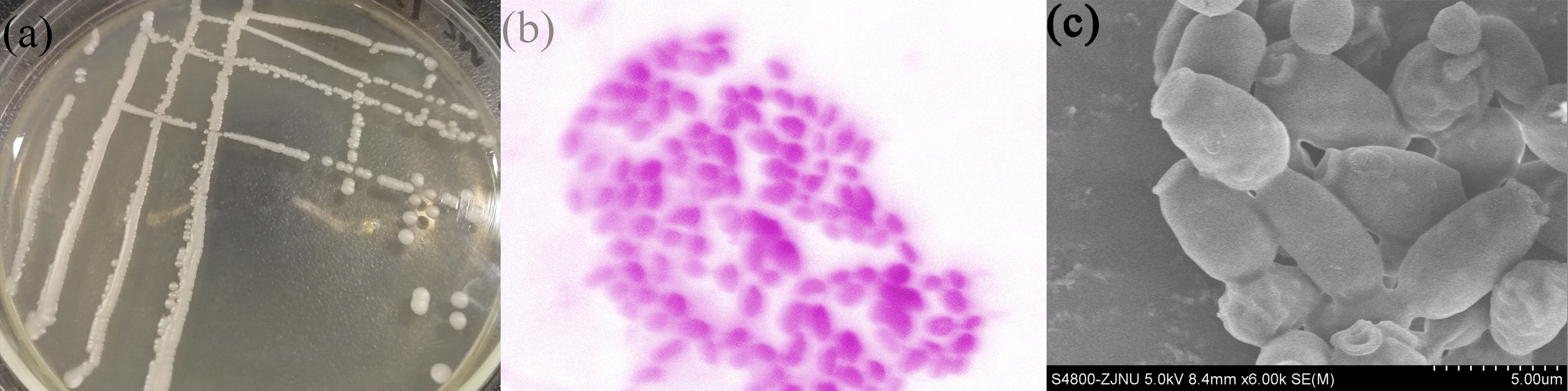


**Fig. S1**. Morphological characteristics of the strain SASS1, (a) colony morphology (b) Gram stain images (c) SEM images.





**Fig. S2**. Cell growth of the strain SASS1 under different culture conditions after 48 h of incubation. (A) Temperature varied from 5 °C to 40 °C; (B) pH varied from 2.0 to 10.0 under pH 4.0; (C) Salt-tolerance ability under optimized temperature and pH;

**References**

Acikgoz, E., and Ozcan, B. (2016). Phenol biodegradation by halophilic archaea. *Int. Biodeter. Biodegr.* 107**,** 140-146. doi: 10.1016/j.ibiod.2015.11.016

Felshia, S.C., Karthick, N.A., Thilagam, R., Chandralekha, A., Raghavarao, K.S.M.S., and Gnanamani, A. (2017). Efficacy of free and encapsulated *Bacillus lichenformis* strain SL10 on degradation of phenol: A comparative study of degradation kinetics. *J. Environ. Manage.* 197**,** 373-383. doi: 10.1016/j.jenvman.2017.04.005

Gomes, M.B., Gonzales-Limache, E.E., Sousa, S.T.P., Dellagnezze, B.M., Sartoratto, A., Silva, L.C.F., et al. (2018). Exploring the potential of halophilic bacteria from oil terminal environments for biosurfactant production and hydrocarbon degradation under high-salinity conditions. *Int. Biodeter. Biodegr.* 126**,** 231-242. doi: 10.1016/j.ibiod.2016.08.014

Ke, Q., Zhang, Y.G., Wu, X.L., Su, X.M., Wang, Y.Y., Lin, H.J., et al. (2018). Sustainable biodegradation of phenol by immobilized *Bacillus* sp SAS19 with porous carbonaceous gels as carriers. *J. Environ. Manage.* 222**,** 185-189. doi: 10.1016/j.jenvman.2018.05.061

Kobayashi, F., Maki, T., and Nakamura, Y. (2012). Biodegradation of phenol in seawater using bacteria isolated from the intestinal contents of marine creatures. *Int. Biodeter. Biodegr.* 69**,** 113-118. doi: org/10.1016/j.ibiod.2011.06.008

Liu, Z.H., Xie, W.Y., Li, D.H., Peng, Y., Li, Z.S., and Liu, S.S. (2016). Biodegradation of phenol by bacteria strain *Acinetobacter Calcoaceticus* PA isolated from phenolic wastewater. *Int. J. Environ. Res. Public Health* 13(3)**,** 300. doi: 10.3390/ijerph13030300

Lu, Z.Y., Guo, X.J., Li, H., Huang, Z.Z., Lin, K.F., and Liu, Y.D. (2015). High-throughput screening for a moderately halophilic phenol-degrading strain and its salt tolerance response. *Int. J. Mol. Sci.* 16(6)**,** 11834-11848. doi: 10.3390/ijms160611834

Nie, H.Y., Nie, M.Q., Yang, Y.Z., Zhao, J., Zhang, X.Y., Guo, Y.T., et al. (2016). Characterization of phenol metabolization by *P*. *stutzeri* N2. *Polycyclic Aromat. Compd.* 36(5)**,** 587-600. doi: 10.1080/10406638.2015.1033434

Senthilvelan, T., Kanagaraj, J., Panda, R.C., and Mandal, A.B. (2014). Biodegradation of phenol by mixed microbial culture: an eco-friendly approach for the pollution reduction. *Clean Technol. Environ. Policy* 16(1)**,** 113-126. doi: 10.1007/s10098-013-0598-2

Shahryari, S., Zahiri, H.S., Haghbeen, K., Adrian, L., and Noghabi, K.A. (2018). High phenol degradation capacity of a newly characterized Acinetobacter sp SA01: Bacterial cell viability and membrane impairment in respect to the phenol toxicity. *Ecotox. Environ. Safe.* 164**,** 455-466. doi: 10.1016/j.ecoenv.2018.08.051

Tian, M.Y., Du, D.Y., Zhou, W., Zeng, X.B., and Cheng, G.J. (2017). Phenol degradation and genotypic analysis of dioxygenase genes in bacteria isolated from sediments. *Braz. J. Microbiol.* 48(2)**,** 305-313. doi: 10.1016/j.bjm.2016.12.002

Van Dexter, S., and Boopathy, R. (2018). Biodegradation of phenol by *Acinetobacter tandoii* isolated from the gut of the termite. *Environ. Sci. Pollut. Res.***,** 1-6. doi: 10.1007/s11356-018-3292-4

Wang, L.B., Xue, C.Z., Wang, L., Zhao, Q.Y., Wei, W., and Sun, Y.H. (2016). Strain improvement of *Chlorella* sp for phenol biodegradation by adaptive laboratory evolution. *Bioresour. Technol.* 205**,** 264-268. doi: 10.1016/j.biortech.2016.01.022

Wu, L.Y., Ali, D.C., Liu, P., Peng, C., Zhai, J.X., Wang, Y., et al. (2018). Degradation of phenol via ortho-pathway by *Kocuria* sp. strain TIBETAN4 isolated from the soils around Qinghai Lake in China. *Plos One* 13(6)**,** e0199572. doi: org/10.1371/journal.pone.0199572

Zhou, W.g., Guo, W.b., Zhou, H.b., and Chen, X.h. (2016). Phenol degradation by *Sulfobacillus acidophilus* TPY via the meta-pathway. *Microbiol. Res.* 190**,** 37-45. doi: 10.1016/j.micres.2016.05.005
